# Stressing out the Hippo/YAP signaling pathway: toward a new role in Schwann cells

**DOI:** 10.1038/cddis.2015.291

**Published:** 2015-10-15

**Authors:** S Melfi, A Colciago, G Giannotti, V Bonalume, L Caffino, F Fumagalli, V Magnaghi

**Affiliations:** 1 Dipartimento Scienze Farmacologiche e Biomolecolari, Università degli Studi di Milano, Via G. Balzaretti 9, Milan, Italy

Throughout the last 15 years, the Hippo pathway has been shown to be one of the most intriguing signaling pathway found to fulfil a crucial role in regulating multiple cellular functions, such as proliferation, apoptosis, regeneration and organ size control. This pathway was first identified in *Drosophila melanogaster*, but it was primarily investigated in tumorigenesis, given its relevance also for human cancer.^1^ Hippo has been conserved during evolution, and accumulating studies supported its tumorigenic involvement during mammal development.^2, 3^ Central modulators of Hippo pathway are the transcriptional coactivators YAP and TAZ, which shuttle between the cytoplasm and the nucleus. Noteworthy, when Hippo is activated, YAP and TAZ are phosphorylated and retained in the cytoplasm, so that their downstream genes are inactivated. Conversely, when Hippo is inactive, YAP and TAZ translocate into the nucleus, enabling the transcription of target genes involved in cell proliferation, survival and tumorigenesis.^3^

Merlin is a cytoskeleton-associated protein belonging to the ERM (ezrin-radixin-moesin) family, which serves as tumor-suppressor protein in different cells. Mutations in the neurofibromin gene (*Nf2*), encoding merlin, are associated with the autosomal dominant multiple syndrome called neurofibromatosis type 2.^4^ Merlin is an upstream activator of Hippo,^5^ and currently, *Nf*2 is considered the only gene of the Hippo pathway that is found to be inactivated via a mutation in cancer, as for example, in vestibular schwannoma.

Recently, a manuscript by K-L Guan *et al.*^6^ reviewed the emerging functions of Hippo, focusing on a new role of YAP and TAZ in cancer progression. They pointed to extracellular factors, mechanotransduction and cell-to-cell adhesion mechanisms as YAP and TAZ regulators. Furthermore, the capability of Hippo/YAP in controlling stem cell renewal and expansion was also demonstrated in mammals and humans.^5, 7^

The complexity of Hippo/YAP pathway increased considerably in the recent years, and other tissues and cells, for instance those forming the nervous system, were proved to be an interesting target of investigation. In vertebrates, YAP may regulate neural progenitor cell number during the central nervous system development.^8^ Therefore, YAP has been appointed as one of the master regulator of neural, as well as glial, progenitor cells in the central nervous system. Hitherto, very few studies tried to elucidate the Hippo/YAP involvement in controlling the biological processes in neural and glial cells of the peripheral nervous system.

Now, we published in *Cell Death and Discovery* that the Hippo/YAP intracellular signaling pathway has an important role in regulating some biological processes in the main glial cells of the peripheral nervous system, the Schwann cells (SC), see Figure 1.^9^ Our findings also corroborated the hypothesis that merlin is involved in modulating the Hippo pathway.^5^ In the manuscript, Colciago *et al.* showed that some genes, known to be upstream or downstream mediators of Hippo (for instance, Amotl2, Dchs, Fat or Wnt1) were changed in SC exposed to an environmental challenge, such as a 50-Hz electromagnetic field.^9^ It is generally known that YAP is also implicated in the Crb/Amotl tight junction signaling serving as tumor suppressor to limit the adhesion-related cell growth.^10^ We found that, in SC, the dysregulation of the Hippo/YAP pathway, produced YAP downregulation, increased cell proliferation and decreased differentiation. In summary, we showed that a cascade of coordinated mechanisms, involving tight junction alterations, decrease in merlin levels, SC migration, increase of the chemo-responsivity, YAP downregulation and redistribution, altogether reduce Hippo pathway activation in SC. This, in turn, may participate in controlling the fate of SC following an environmental challenge.^9^ Indeed, some recent data suggested that YAP, besides its oncogenic potential in several cancers, might also display tumor-suppressor functions in different cellular context. Therefore, the observed changes in cellular distribution and the augmented availability of YAP in the SC cytoplasm, as we proposed,^9^ might account for its capability to induce cell oncogenic transformation.

The interest of researchers in Hippo/YAP proteins in SC development and myelination is recently raising, as witnessed by the data from Parkinson and co-workers.^11^ They showed that this pathway is likely activated following the loss of merlin in SC, causing a large increase in SC proliferation and defects in axonal regeneration.^11^

Moreover, novel insights on the importance of Hippo/YAP signaling in the peripheral nervous system have been provided by Serinagaoglu *et al.*^12^ These authors described how YAP and TAZ are expressed in migratory neural crest cells, in dorsal root ganglion (DRG) progenitors and peripheral glial cells (mostly satellite glial cells), but not in neuronal lineage. YAP was inhibited by merlin during normal DRG development, while the loss of *Nf2* led to YAP hyperactivation, overexpansion of DRG progenitors and glial cells, concomitantly to a reduction of neuron development.^12^ These findings supported a YAP-mediated signaling during neural crest development. Unraveling these mechanisms may have important future clinical implications.

The environmental control of Hippo/YAP might have a relevant socioeconomic impact. The low-frequency (20 Hz) electromagnetic fields have long been proposed as therapy to promote peripheral nerve regeneration,^13^ whereas the 50-Hz electromagnetic fields are largely used in several medical tools and in common electric devices, for instance, the mobile phones. However, the risk to develop vestibular schwannomas, coming from the long-term use of wireless phones, is still unclear and debated, although clinical outcomes seem to corroborate this pathogenic correlation.^14^ Further studies are needed to support this hypothesis, but people should be aware that these scientific issues are open and very actual, and should be carefully considered for human health.

## Figures and Tables

**Figure 1:**
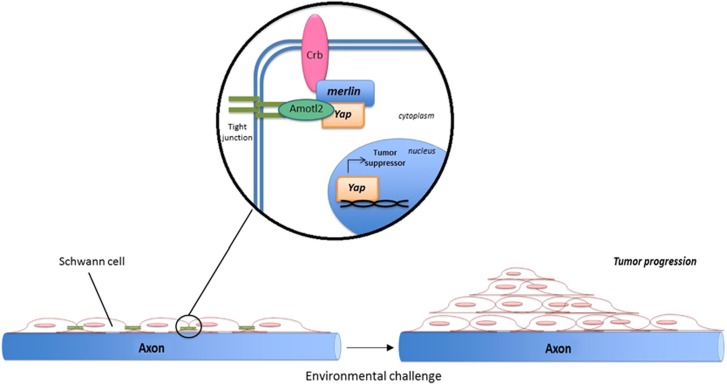
Model of Hippo/YAP involvement in Schwann cells of the peripheral nervous system. Hippo/YAP participate in forming tight junction and cell-to-cell adhesion in Schwann cells. A potential injury or an environmental challenge, as the exposure to a 50-Hz electromagnetic field, may induce a merlin-dependent Hippo/YAP activation, in turn affecting cell proliferation, migration, chemotactic responsivity and differentiation. In principle, these mechanisms are supposed to be involved in nerve tumorigenesis and schwannoma development^9^
